# Epidemiology of *Haemophilus influenzae* Serotype a, North American Arctic, 2000–2005

**DOI:** 10.3201/eid1401.070822

**Published:** 2008-01

**Authors:** Michael G. Bruce, Shelley L. Deeks, Tammy Zulz, Christine Navarro, Carolina Palacios, Cheryl Case, Colleen Hemsley, Tom Hennessy, Andre Corriveau, Bryce Larke, Isaac Sobel, Marguerite Lovgren, Carolynn DeByle, Raymond Tsang, Alan J. Parkinson, the International Circumpolar Surveillance Hia Working Group

**Affiliations:** * Centers for Disease Control and Prevention, Anchorage, Alaska, USA; † Public Health Agency of Canada, Ottawa, Ontario, Canada; ‡ Nunavut Department of Health, Iqaluit, Nunavut, Canada; § Northwest Territories Department of Health and Social Services, Yellowknife, Northwest Territories, Canada; ¶ Yukon Health and Social Services, Whitehorse, Yukon, Canada; # National Centre for Streptococcus, Edmonton, Alberta, Canada; ** National Microbiology Laboratory, Winnipeg, Manitoba, Canada

**Keywords:** Haemophilus influenzae, Hia, Hib, emerging infections, indigenous, surveillance, Alaska, Canada, meningitis, pneumonia, septic arthritis, research

## Abstract

Before the introduction of *Haemophilus influenzae* type b (Hib) conjugate vaccines, rates of invasive *H. influenzae* disease among indigenous people of the North American Arctic were among the highest in the world. Routine vaccination reduced rates to low levels; however, serotype replacement with non–type b strains may result in a reemergence of invasive disease in children. We reviewed population-based data on invasive *H. influenzae* in Alaska and northern Canada from 2000–2005; 138 cases were reported. Among 88 typeable isolates, 42 (48%) were *H. influenzae* type a (Hia); 35 (83%) occurred in indigenous peoples. Among Hia patients, median age was 1.1 years; 62% were male; 1 adult died. Common clinical manifestations included meningitis, pneumonia, and septic arthritis. Overall annual incidence was 0.9 cases per 100,000 population. Incidence among indigenous children <2 years of age in Alaska and northern Canada was 21 and 102, respectively. Serotype a is now the most common *H. influenzae* serotype in the North American Arctic; the highest rates are among indigenous children.

## Abstract 2

Serotype a is now the most common seen in the North American Arctic; highest rates occur in indigenous children.

*Haemophilus influenzae* causes illnesses ranging from local respiratory infection to serious invasive disease, including meningitis, epiglottitis, septic arthritis, and septicemia (*1*). Among the encapsulated strains (a to f) that have been identified, *H. influenzae* serotype b (Hib) is the most virulent (*1*–*4*). Nonencapsulated (nontypeable) strains are usually associated with noninvasive infections but can cause invasive disease, including neonatal sepsis (*2*–*5*). Historically, Hib was the leading cause of bacterial meningitis in the United States and Canada (*6*). However, since the introduction of Hib capsular polysaccharide-protein conjugate vaccine in 1988, the incidence of invasive Hib disease has declined dramatically. Data from the Centers for Disease Control and Prevention (CDC) show invasive Hib disease in the United States has decreased by 99% to <1 case per 100,000 children <5 years of age (*7*). Similar declines have been documented in Canada (*8*).

Indigenous people, defined as the original people of Alaska (Alaska Native people) and northern Canada (aboriginal people), are at increased risk for Hib disease (*8*–*12*) than the general populations of the United States and Canada, and the risk for disease peaks at an earlier age (*12*–*14*). While Hib vaccination led to the rapid decline of Hib disease in all populations including indigenous groups, indigenous children continue to have higher rates of Hib disease than nonindigenous children (*12*,*15*).

With widespread vaccination against Hib, concern has been raised about the potential for replacement disease caused by non–type b encapsulated strains. Protection conferred by the Hib vaccine is specific to the type b polysaccharide capsule. It was suggested that reducing carriage of the vaccine type may open an ecologic niche, allowing increased colonization with non–type b strains of *H. influenzae* with the potential to become invasive (*6*,*16*).

Non–type b *H. influenzae* disease is uncommon in children; however, since the introduction of Hib vaccine, the relative importance of infections due to nonencapsulated and non–type b encapsulated *H. influenzae* has increased (*3*). Infections caused by nonencapsulated strains are more common in adults and are more likely to be associated with pneumonia, whereas infections caused by encapsulated strains tend to occur in younger children with a predominance of meningitis and bacteremia (*3*,*17*). Non–type b *H. influenzae* appears to be more common in persons with underlying medical illnesses, such as immunosuppressive conditions (*3*,*4*,*17*). The extent to which non-b *H. influenzae* causes invasive disease is not fully known (*17*). In some countries, only Hib disease is reportable; therefore, information on other serotypes is lacking. However, numerous case reports of invasive *H. influenzae* disease caused by encapsulated non-b serotypes, particularly types a, e, and f, have been published (*6*,*10*,*11*,*18*–*20*).

Although uncommon, *H. influenzae* serotype a (Hia) has been reported to cause invasive disease, meningitis, pneumonia, and sepsis (*3*,*20*). Hia disease may occur more frequently in indigenous populations (*10*,*11*,*21*). Reports of invasive *H. influenzae* disease have identified Hia in 7.8% of Australian aboriginal children and 16.7% of Apache children with invasive disease (*10*,*21*–*23*). The Navajo and White Mountain Apache populations have a higher rate of Hia disease than the general US population, and Hia is now a leading cause of invasive *H. influenzae* disease in these populations (*10*). Although high rates were discovered during the period of surveillance (1988–2003), significant increases in incidence were not found (*10*). Seasonal, temporal, and geographic clustering was not demonstrated. An outbreak of invasive Hia has recently been described in Alaska (*11*), and data from the International Circumpolar Surveillance (ICS) Program suggest that the number of cases of Hia has increased in both Alaska and northern Canada. The objectives of this study were to characterize cases of invasive Hia, to examine incidence rates over time, and to assess the relatedness of Hia isolates by molecular typing.

## Methods

ICS, a population-based surveillance system for invasive bacterial diseases established in 1999, includes laboratory-based surveillance for *Streptococcus pneumoniae*, *H. influenzae*, *Neisseria meningitidis*, and groups A and B streptococci. Current member countries include the US Arctic (Alaska), northern Canada, Greenland, Iceland, Norway, northern Sweden, and Finland.

This study reviews data collected from 2000 through 2005 from Alaska and northern Canada. In Alaska, 23 laboratories throughout the state are asked to send any isolate of *H. influenzae* recovered from a normally sterile site to a reference laboratory in Anchorage at CDC’s Arctic Investigations Program, which serves as the data repository for ICS. In northern Canada, a network of laboratories within the regions (Yukon, Northwest Territories, Nunavut, northern Quebec, and northern Labrador) participate, as well as 3 reference laboratories (2 national and 1 provincial). The laboratories are requested to send any isolate of *H. influenzae* recovered from a normally sterile site to the appropriate reference laboratory to confirm the identity, determine the serotype, and test for antimicrobial drug susceptibility. Laboratory, demographic, and clinical data are collected for each invasive case of *H. influenzae* occurring in Alaska and northern Canada, and these data are forwarded to ICS headquarters in Alaska.

A case of invasive *H. influenzae* disease is defined as illness in a resident of the surveillance area from whom *H. influenzae* is isolated from a sample obtained from a normally sterile site, including blood, cerebrospinal fluid, pleural fluid, peritoneal fluid, or joint fluid. Patients with clinical epiglottitis from which *H. influenzae* is isolated from an epiglottis swab are also reportable to ICS. The primary clinical manifestation of *H. influenzae* was determined by a review of the patient’s medical record.

Population denominator data for Alaska and northern Canada were obtained from the Alaska Department of Labor and Workforce Development (www.labor.state.ak.us), Statistics Canada (www.statcan.ca), and the Demography Division of Statistics Canada. Estimates from Alaska and northern Canada reflect population figures from the 2000 and 2001 census years, respectively. Canadian indigenous estimates were calculated by using population data from the Aboriginal Population Profile, which is developed from 2001 Census data. This study covers a 6-year surveillance period, January 1, 2000–December 31, 2005. Alaska and northern Canada’s estimated populations were 655,435 and 132,956, respectively. Indigenous peoples comprised 19% of the population in Alaska and 59% of the population in northern Canada.

### Laboratory Methods

Isolates were streaked onto chocolate agar to check for purity and confirmed to be *H. influenzae*. Confirmation tests included a requirement for both X (hemin) and V (nicotinamide adenine dinucleotide) growth factors (Oxoid, Hampshire, UK), Gram stain, and serotyping by slide agglutination.

### Antimicrobial Susceptibility Testing

Susceptibility testing in Alaska was performed by using Etest (AB Biodisk, Solna, Sweden). A direct colony suspension equivalent to a 0.5 MacFarland standard was prepared in Mueller-Hinton broth from an overnight culture. *Haemophilus* Test Medium (Remel, Lexna, KS, USA) was added to produce a confluent lawn of growth, and then Etest strips were placed onto the plate. The plates were then incubated for 20–24 hours at 35°C in 5% CO_2_. The MIC was read at the point of intersection of growth and the strip. Susceptibility of *H. influenzae* to the following antimicrobial drugs was tested: ampicillin, ceftriaxone, meropenem, chloramphenicol, and trimethoprim-sulfamethoxazole (TMP-sulfa). Susceptibility data were not available from Canada.

### Serotyping

Capsular serotyping was performed by slide agglutination with Difco antisera (Difco, Detroit, MI, USA) in Alaska and Remel antisera (Remel Europe Ltd, Dartford, UK) in northern Canadian laboratories (with the exception of the Laboratoire de Sante Publique in Quebec, which used PCR). If no capsular polysaccharide was present, the isolate was classified as nontypeable by slide agglutination. Known positive and negative controls were run weekly, and each culture was screened in saline alone to check for autoagglutination. Since 2005, laboratories have participated in an ongoing *H. influenzae* quality control program.

### Pulsed-field Gel Electrophoresis (PFGE) and PCR

A selection of invasive isolates of Hia from cases in Alaska and northern Canada that tested positive for serotype a by both slide agglutination and genotype-specific PCR (*24*) were examined by PFGE with the use of the restriction enzymes *Sma*I and *Apa*I digestions (*18*,*25*) at the CDC laboratory in Anchorage, Alaska. The fragments were resolved with a CHEF DRII (Bio-Rad, Hercules, CA, USA) (2.2- to 35-s switch times) at 175V for 21 hours. DNA banding patterns were analyzed with BioNumerics version 3.0 software (Applied Maths, Sint-Martens-Latem, Belgium). Percentage similarities were identified on a dendrogram derived from the unweighted pair group method by using Dice coefficients and a band position tolerance of 1.5%. A similarity coefficient of 80% (<3-band difference) was used to define related groups. The IS*1016*-bexA deletion was amplified from genomic DNA by PCR using sense IS*1016* (5′-ATTAGCAAGTATGCTAGTCTAT-3′) and antisense bexA (5′-CAATGATTCGCGTAAATAATGT-3′) primers (*26*).

### Statistical Analysis

Data were double entered into Paradox v9.0 (Corel, Ottawa, Ontario, Canada), and analyzed by using Epi Info version 6.04b (CDC, Atlanta, GA, USA) and StatXact version 6.2 (CYTEL Software Corp., Cambridge, MA, USA). Statistical differences in rates between periods and between countries were assessed by using a 2-sample Poisson test; p values are exact when appropriate.

## Results

### Descriptive Epidemiology

**Figure 1:**
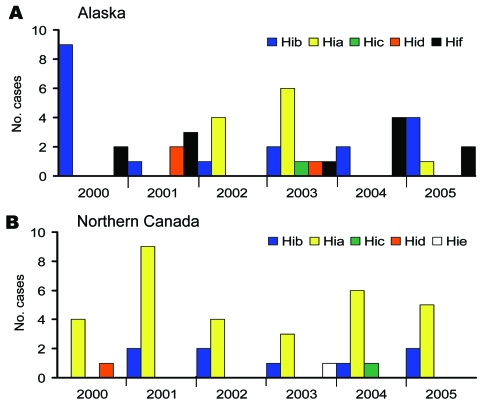
*Haemophilus influenzae* (Hi) cases by serotype in Alaska and Northern Canada, 2000–2005. A) Alaska; n = 46 typeable (27 nontypeable); 59% encapsulated non-b, 24% Hia. B) Northern Canada; n = 42 typeable (17 nontypeable); 81% encapsulated non-b, 74% Hia.

We identified 138 cases of invasive *H. influenzae* disease from 2000 through 2005; serotype data were available for 132 (96%) of the isolates. Among these, 44 (33%) were nontypeable (Alaska 27; northern Canada 17). Of the remaining 88 isolates, 42 (48%) were serotype a, 27 (31%) serotype b, 12 (14%) serotype f, 4 (5%) serotype d, 2 (2%) serotype c, and 1 (1%) serotype e (Figure 1). The proportion of illnesses that resulted in death among Hia, encapsulated non-a, and nontypeable isolates was 5% (2/37), 14% (6/42), and 15% (6/40), respectively.

Among the 42 Hia isolates, 30 (71%) occurred in children <2 years of age; 4 (10%) occurred in children 2–5 years of age; the remaining 8 (19%) occurred in adults (range 21–73 years). Ethnicity data were available for 38 cases; 35 (92%) occurred in indigenous people. The median age among case-patients was 1.1 years (range 3 months to 73 years) and did not differ significantly by country. Overall, 62% were male (Table 1). Cases occurred in 3 Alaska regions and 3 regions in northern Canada. Most cases (60%) occurred in 1 northern Canadian region. No clear seasonal pattern of invasive Hia disease was observed; however, 5 (50%) cases of invasive Hia that occurred in indigenous Alaska children <2 years of age were clustered over a 5-month period in 2003 and occurred in 2 villages in western Alaska (*11*). No clusters >2 cases in 1 village over a period of 2 months were observed in northern Canada. No pattern of increasing incidence rates was seen over the study period.

**Table 1:** Characteristics of Alaskan and northern Canadian persons with invasive *Haemophilus influenzae* type a disease, 2000–2005

Characteristic	Alaska (n = 11)	Northern Canada (n = 31)	Total (n = 42)
Median age (range)	10 mo (4 mo–73 y)	1.2 y (3 mo–31 y)	1.1 y (3 mo–73 y)
Male sex, no. (%)	7 (64)	19 (61)	26 (62)
Indigenous, no. (%)	8 (73)	27 (100)*	35 (92)
Age appropriately vaccinated for Hib, y (% vaccinated)	7 (88)†	21 (88)‡	28 (88)
Hospitalization, no. (%)‡	10 (91)	25 (96)§	35 (97)
Deaths, no. (%)¶	1 (9)	1 (4)	2 (5)

*Ethnicity data missing from 4 cases; denominator = 27. †Vaccine history missing from 3 cases; denominator = 8. ‡Vaccine history missing from 7 cases; denominator = 24. §Hospitalization data missing from 5 cases; denominator = 26. ¶Death data missing from 5 cases; denominator = 26.

### Incidence Rates by Age

Overall crude annualized incidence of invasive Hia for the 6-year study period was 0.9 cases per 100,000 population. In Alaska and northern Canada, crude annualized incidence rates were 0.3 and 3.9 cases per 100,000 population, respectively. Annualized incidence rates of invasive Hia in children <2 years of age were 19.7 cases per 100,000 population; annualized rates in Alaskan and northern Canadian children <2 years were 5.7 and 79.1 cases per 100,000 population, respectively (p<0.001, Table 2).

**Table 2:** Cases and annualized incidence rate per 100,000 population of Invasive *Haemophilus influenzae* type a disease by age and ethnicity, 2000–2005*

Demographic group	Total no. (range/y); rate (range/100,000/y)	Alaska, no. cases (rate)	Northern Canada, no. cases (rate)	p value*
Overall	42 (4–9); 0.9 (0.5–1.2)	11 (0.3)	31 (3.9)	<0.001
<2 y of age	30 (1–8) ;19.7 (4.0–31.4)	7 (5.7)	23 (79.1)	<0.001
Indigenous	35 (4–8); 2.9 (2.0–4.0)	8 (1.1)	27 (5.9)	<0.001
<2 y of age, indigenous	29 (1–8); 52.6 (11.2–86.2)	7 (20.9)	22 (101.9)	<0.001

*p value for rate, Alaska vs. northern Canada.

### Incidence Rates by Ethnicity

The overall annualized crude incidence rates of invasive Hia over the study period in indigenous and nonindigenous people were 2.9 and 0.2 cases per 100,000 population, respectively. Among indigenous people, the overall annual crude incidence rates ranged from 2.0 to 4.0 cases per 100,000 population during the study period; annualized indigenous incidence rates in Alaska and northern Canada were 1.1 and 5.9 cases per 100,000 population, respectively (Table 2). Among indigenous children <2 years of age, the overall annualized incidence rate was 52.6 cases per 100,000 population, 20.9 and 101.9 cases per 100,000 persons in Alaska and northern Canada, respectively (Table 2).

### Clinical Illness

Among all ages, the most common clinical syndromes were meningitis (33%) and pneumonia (29%), followed by septic arthritis (12%). Clinical manifestations differed between children and adults. As noted previously, all pediatric cases occurred in children <5 years of age. Adult case-patients were 21–73 years of age. Children were more likely to exhibit meningitis, and adults were more likely to exhibit pneumonia; septic arthritis was reported among 5 (15%) pediatric patients. Clinical features were similar in Alaska and northern Canada. No cases of epiglottitis were reported (Table 3).

**Table 3:** Clinical illness in Alaskan and northern Canadian children and adults with invasive *Haemophilus influenzae* type a disease, 2000–2005*

Diagnosis	Alaska (n = 11)			Northern Canada (n = 31)			North American Arctic (n = 42)	
	Children (n = 7), no. (%)	Adults (n = 4), no. (%)		Children (n = 27), no. (%)	Adults (n = 4), no. (%)		Children (n = 34), no. (%)	Adults (n = 8), no. (%)
Meningitis	2 (29)	0		12 (44)	0		14 (41)	0
Pneumonia	3 (43)	3 (75)		4 (15)	2 (50)		7 (21)	5 (62)
Bacteremia	0	1 (25)		6 (22)	1 (25)		6 (18)	2 (25)
Septic arthritis	2 (29)	0		3 (11)	0		5 (15)	0
Other†	0	0		2 (8)	1 (25)		2 (3)	1 (13)

*Children, <18 years of age; adults, >18 years of age. †Includes osteomyelitis (1 child), pericarditis (1 adult), and cellulitis (1 child).

Of the 37 case-patients with known hospitalization status, 35 (95%) were hospitalized with a median duration of 8 days. Case-patients in Alaska had a shorter median duration of hospitalization than those in northern Canada (6.5 vs. 9.0 days). Outcome information was available for 37 cases. Two patients (1 indigenous child, 1 nonindigenous adult) with invasive Hia died (Table 1). Both patients who died were diagnosed with pneumonia; neither had a history of immunodeficiency noted in the chart.

### Recurrence

Two Alaskan Hia patients had recurrent disease. In 1 patient, the clinical manifestation was septic arthritis in both occurrences (4 months before recurrence). In the second patient, the initial clinical syndrome was pneumonia, followed by meningitis 4 months later. Both of these patients were <1 year of age when first brought for treatment. Neither child had documented immunodeficiency (*11*). No recurring cases of Hia were documented in northern Canada.

### Antimicrobial Susceptibility Testing

All 11 Hia isolates from Alaska were susceptible to ampicillin, ceftriaxone, meropenem, and chloramphenicol. Ten of 11 Hia isolates from Alaska were tested for TMP-sulfa resistance; 2 of the 10 (20%) demonstrated intermediate resistance.

### PFGE and PCR analysis

**Figure 2:**
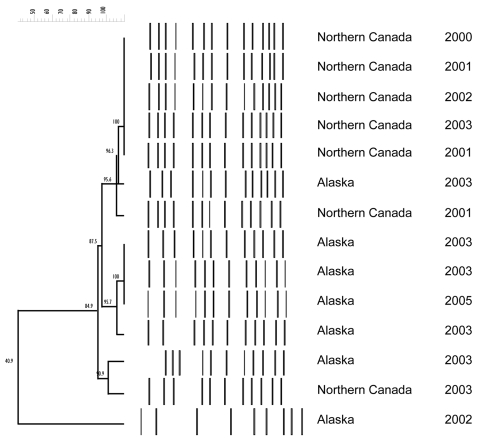
Pulsed-field gel electrophoresis of representative *Haemophilus influenzae* serotype a isolates from Alaska and Northern Canada, 2000–2005 (N = 14).

PFGE was performed on 9 Hia isolates from Alaska and 19 isolates from northern Canada. With 1 exception, all isolates were found to be closely related (<3-band difference) with a Dice correlation of >85% (Figure 2). All 28 isolates were negative for the IS*1016-bexA* deletion by PCR.

## Discussion

Our data demonstrate that 69% of invasive *H. influenzae* disease in the North American Arctic is now caused by non-b serotypes (Alaska 51%; northern Canada 89%) with serotype a comprising almost half of cases (Alaska 24%; northern Canada 74%). Hia is now the most prevalent serotype in the North American Arctic. The clinical features of invasive Hia cases were similar to those of invasive disease caused by Hib. The overall annualized incidence of Hia in children <2 years of age living in the North American Arctic was 19.7 cases per 100,000 children (Alaska 5.7; northern Canada 79.1) in contrast to the rate of 0.83 cases of non–type b invasive *H. influenzae* disease among US children <5 years of age (*7*). The overall annualized incidence rate among indigenous children <2 years of age residing in the North American Arctic was particularly high at 52.6 cases per 100,000 children (Alaska 20.9; northern Canada 101.9).

Widespread use of Hib conjugate vaccine has resulted in a dramatic decline in invasive Hib disease in the United States and Canada (*7*,*8*), including among indigenous children in the North American Arctic (*7*,*8*,*14*) and the southwestern United States (*12*,*27*). However, the potential for serotype replacement remains a concern (*16*). Three population-based studies have documented small increases in the incidence of non–type b *H. influenzae* disease after the introduction of the Hib conjugate vaccine (*28*–*30*). Several recent studies conducted in different countries have demonstrated a predominance of serotype a disease. A study by Tsang et al. in Manitoba, Canada, found that most of the 52 *H. influenzae* cases reported during 2000–2004 were caused by serotype a (50%) or nonserotypeable isolates (38.5%) (*31*). Ribeiro et al. noted an 8-fold increase in the incidence of Hia meningitis from the prevaccine (0.02 per 100,000 population) to postvaccine period (0.16 per 100,000 population) in Brazil (*32*). However, more recently published data by this group demonstrate that the increase in incidence during the year following vaccine introduction was not observed in subsequent years (*33*). A study by Millar et al. has demonstrated high rates of invasive Hia disease among Navajo and White Mountain Apache children in the southwestern United States, although no increase in Hia incidence was noted after the introduction of Hib vaccine (*10*).

Clinical manifestations of invasive Hia disease were similar to those of invasive Hib disease in the prevaccine era (*29*). Meningitis was more common among Hia infections than in infections caused by encapsulated non-a and non-typeable Hi infections (37% vs. 24% and 12%, respectively, p = 0.05); conversely, pneumonia was less common (32% Hia vs. 41% encapsulated non–a *H. influenzae* and 40% nontypeable *H. influenzae*); however, this finding was not statistically significant. Clinical disease varied with age when treatment was sought; meningitis was more common in children, and bacteremic pneumonia was more common in adults. The differing age distribution of Hia patients compared to patients with non-a and nontypeable infections may account for the differing clinical illnesses. Invasive Hia disease tended to occur among young children and nontypeable *H. influenzae* infections among adults (median age 1.1 vs. 39.2 years, respectively, p = 0.0003). The higher median age of patients infected with nontypeable *H. influenzae* is consistent with results of a recent US study that demonstrated an increase in the number of cases of invasive nontypeable *H. influenzae* among adults (*30*) in the postvaccine era, and a study by McVernon et al. in England and Wales that showed an increase in invasive non-b *H. influenzae* among older age groups (*34*).

There are several possible explanations for the high proportion of Hia among invasive *H. influenzae* disease in the surveillance population. First, an increase in virulence might explain the current predominance of serotype a. The IS*1016-bexA* deletion enables production of more capsule, which is thought to be the major virulence factor for invasive disease (*26*,*35*). However, Hia isolates from Alaska or northern Canada tested negative for the *bexA* deletion, and it appears unlikely that the high rates of invasive Hia disease, particularly among indigenous children in this region, are due to introduction of a particularly virulent strain of Hia. If a new highly virulent Hia strain were introduced into the North American Arctic in the postvaccine period, subtyping data may show a clonally restricted pattern. We found a high degree of relatedness with a predominance of 1 clone across the North American Arctic. However, these data do not directly support the introduction of a virulent clone because other studies suggest limited genetic diversity of Hia (*18*,*36*). Second, widespread use of Hib conjugate vaccine and the subsequent reduction in Hib colonization may have opened an ecologic niche for increased colonization with Hia or other non-Hib strains. Little data regarding carriage of non–type b strains of *H. influenzae* are available; however, an investigation of a cluster of 5 of the invasive Hia cases in Alaska found that among 31 close contacts of case-patients, 5 (16%) were colonized with Hia. Two of the 3 case-patients were infants with recurrent Hia disease; reexposure is the likely explanation for disease recurrence (*11*). Finally, a preexisting background rate of non-b serotype disease may have simply been uncovered due to the decreasing Hib rates. Further studies of invasive *H. influenzae* disease are needed to describe clinical and epidemiologic features, characterize the pattern and rates of colonization, determine risk factors for carriage, and further characterize the strains by using molecular techniques.

Hia disease raises many questions from a public health response perspective. While chemoprophylactic regimens are well described for contacts of persons with Hib disease (*37*), the utility of chemoprophylaxis or other public health prevention measures for non-b typeable disease such as Hia is not clear. Further research is needed to provide clear guidance to practicing physicians caring for patients with Hia disease.

This study has several limitations. Alaskan and northern Canadian data on non-b invasive *H. influenzae* disease were not collected in the pre-Hib conjugate vaccine era, making it difficult to determine baseline or prevaccine incidence of serotype a in this region. We did not collect detailed clinical and demographic information beyond what was available from medical record review, and therefore we were not able to assess other factors (e.g., *H. influenzae* carriage among case-patients, within the community, or among close contacts). In addition, most *H. influenzae* isolates are serotyped by using slide agglutination only. Because PCR is not yet routinely used throughout the ICS network for serotyping, nontypeable strains could have been misclassified as encapsulated strains.

This article analyzed population-based surveillance for invasive *H. influenzae* disease across the North American Arctic. We identified a high proportion of non-b serotypes over the 6-year study period, with particularly high rates of invasive disease caused by Hia. The reason for these high rates of invasive Hia disease is unknown and is likely multifactorial. While Hia incidence rates are high among particular groups in the North American Arctic, case numbers remain low (4–9 cases per year) and are lower than Hib rates in the prevaccine era. Hib vaccination remains one of the great public health success stories. Continued surveillance for *H. influenzae* disease is needed; however, to identify emerging problems and provide data necessary to develop effective prevention strategies.

The changing epidemiology of invasive *H. influenzae*disease highlights the importance of continued surveillance for invasive *H. influenzae* disease in regions of the world where Hib conjugate vaccine is currently in use. ICS will continue to monitor invasive disease caused by all *H. influenzae* serotypes in the North American Arctic and other participating Arctic countries.
